# The Effect of Animal-Assisted Therapy on the State of Patients’ Health After a Stroke: A Pilot Study

**DOI:** 10.3390/ijerph16183272

**Published:** 2019-09-06

**Authors:** Kristýna Machová, Radka Procházková, Michal Říha, Ivona Svobodová

**Affiliations:** 1 Department of Ethology and Companion Animal Science, Faculty of Agrobiology, Food and Natural Resources Czech University of Life Sciences, 16500 Prague, Czech Republic; 2 Department of Statistics, Faculty of Economics and Management, Czech University of Life Sciences, 16500 Prague, Czech Republic; 3 Physical Medicine and Rehabilitation, Military University Hospital Prague, Department of Neurosurgery and Neurooncology, Military University Hospital and First Medical Faculty, Charles University, 16500 Prague, Czech Republic

**Keywords:** stroke, animal assisted therapy, rehabilitation, dog

## Abstract

A stroke is a condition that can give rise to consequences such as cognitive and physical constraints, which sometimes manifest in the psychological condition of the patient. Such patients commence rehabilitation as soon as is possible, which involves a multi-disciplinary approach to treatment. One aspect of complementary rehabilitation could be animal-assisted therapy (AAT). A total of 15 individuals were split into an experimental group comprising 6 patients (2 males, 4 females), and a control group of 9 patients (3 males, 6 females). The participants in the control group were aged from 43 to 87 years and the experimental group featured participants aged from 45 to 76 years. Both groups received standard physiotherapy and occupational therapy. In addition, the experimental group was supplemented with AAT, with the animal in question being a dog. The tools primarily applied to measure the outcomes were the Barthel index, blood pressure, and heart rate measurements, whereas the Likert scale was employed to discern the mood of the patients. The results showed that changes in the values for heart rate and blood pressure were insignificant. However, a statistically significant aspect of the research pertained to the patients confirming that they felt better after the AAT sessions. Hence, AAT could potentially bolster the effectiveness of other therapies.

## 1. Introduction

Stroke is reported as the second most frequent medical condition in modern times. The onset of ischemia or hemorrhaging often gives rise to disorders such as paralysis, depression, and aphasia [1,2]. Between 50–60% of sufferers experience a motor-related disability that decreases the quality of life [3]. Naturally, it is imperative that any subsequent rehabilitation commences promptly afterwards, wherein various methods of physical and occupational therapy are applied, alongside speech therapy [4] and psychotherapy [5]. 

Animal-assisted therapy (AAT) shows potential as a supplementary therapy to standard procedures [6]. Studies have revealed that the presence of an animal can reduce the activity of the sympathetic nervous system, thus diminishing the risk of physical and mental stress [7]. Tsai et al. [8] observed that heart rate and blood pressure decreased in the presence of a dog [9,10], as well as the opportunity for decrease in the cortisol blood level [11]. Presence of a dog during therapy could also leads to a reduction in the physical and mental pain subjectively perceived by the patient at a given moment [12]. Moreover, AAT was observed to positively influence patients with depression [13] and those treated for anxiety [8].

It is also recommended to optimize sensory stimuli, improve orientation, and encourage the incidence of visitations by family members in order to aid the rehabilitation of patients after a stroke [14]. Incorporating AAT into their treatment could provide a fresh stimulus and a means of averting as recommended in literature or disrupting a typical stay in hospital, with the added benefits of facilitating the healing process and hastening its progress [15,16]. 

Stroke victims are highly prone to bouts of depression, brought on by their condition and fear for the future [1]. As a consequence of reduced self-sufficiency and mobility [3], any means of motivating such individuals is welcome [16]. Patients receiving AAT have been observed to focus longer on an activity and demonstrate enthusiasm for further endeavors [17]. Moreover, interacting with a dog, particularly walking alongside it, has several benefits, i.e., encouraging correct posture, regaining momentum, and promoting suitable movement in such patients [18].

Animals possess qualities that mark them out as uniquely suited to this role, mainly since the creature is spontaneous, loyal, loving, and readily available for therapy [19]. These inherent traits help to motivate the patient during the process of treatment [20] and enhance their ability to focus during the session. Assuming the necessary hygienic pre-requisites are in place, AAT is safe, with no increased risk of transmitting infectious diseases to humans [21].

Herein, the current research aimed to determine whether supplementing standard therapy with AAT was beneficial to the rehabilitation of patients after a stroke. In a pilot study, we assumed the positive effect, especially in a psychological capacity; any resulting benefit might then contribute toward the patient cooperating further with other therapists. Various studies have reported positive observations in blood pressure, heart rate, and self-sufficiency, i.e., indicators influencing the stress and health of patients. Thus, prior to commencing research, the assumption of the authors was that the group of patients subjected to AAT would show better results (gauged as output measurements) than the control group, for whom no dog was present during treatment.

## 2. Materials and Methods

### 2.1. Participants

The ages of the patients in the experimental group (*n* = 6; 2 males, 4 females) who completed the course of the study ranged from 45 to 76 years (mean 66.66, median 69.0, standard deviation 11.05). The patients in the control group (*n* = 9; 3 males, 6 females) were aged 43 to 87 years (mean 65.11, median 66.0, standard deviation 14.0). All the patients completed the six-week cycle of therapy. 

Every patient expressly agreed to the potential presence of the dog at their therapeutic sessions. Subsequently, they were randomly allocated into the control and experimental groups. Testing procedures and work with patients were carried out by the Ethical Committee of the Military University Hospital Prague. Participation of animal and assicuration on his welfare was approved by the Institutional Review Board of the Czech University of Life Sciences (CULS) in Prague. 

### 2.2. General Procedures

Determinations of diagnosis and applicable treatment and testing were carried out at the Military University Hospital Prague within the Department of Rehabilitation and Physical Medicine on the premises of Military University Hospital Prague. Data collection and the selection process of patients for the study occurred shortly after the patients had been hospitalized at this department. The subsequent period of research lasted six weeks, with all the patients undergoing continuous assessment of their health status and physiological functions, as well as other necessary tests. 

Both groups received standard physiotherapy and occupational therapy (neurophysiological concepts twice a day, robotic rehabilitation, mobility, and daily activity training). In addition, for the experimental group supplemented with AAT, the animal in question was a dog. Prior to the commencement of research, the therapist working with the dog conferred with the occupational therapist/physiotherapist and agreed upon the most suitable content of each session for every member of the experimental group, which also lent the former insight into the statuses of the given patients. It was decided that the dog would spend about twenty minutes twice a week with each individual per session, which included elements for training the memory, speech exercises, and practicing fine/gross motor skills. In addition to these, relaxation and developing a friendly relationship with the dog and therapist formed an essential part of the AAT; everything involved was geared toward the needs of the patient and their state of mind at the time. 

Each member of the cohort consented to participate in the study by signing an agreement approved by the Ethics Committee of the Military University Hospital Prague which contained a detailed review of the study, its objectives, course, and methods. Participation was entirely voluntary and patients were allowed to withdraw from the research program at any time. 

### 2.3. The Therapy Dog 

The therapy of the experimental group was conducted with the participation of a four-year-old border collie named Mia. Prior to her joining the therapy sessions, Mia was trained to be obedient under common and stressful situations. She had been working with her trainer in the hospital regularly, so she was used to interacting with strangers, and she displayed happiness in playing and interacting with patients and healthcare providers. Her training was ended with a final testing and a certification of the dog and the trainer. Notably, Mia showed no aggression toward humans or animals and she was under regular control of a veterinarian doctor. 

Mia’s work was variable, she had plenty of time for rest, she switched between indoor and outdoor activities, and she always had permanent access to water. She was always working with her trainer, who controlled the quantity of food to avoid any overfeeding.

### 2.4. Measurements

The patients were monitored for progress by gauging their physiological parameters, reporting a subjective mood, and through conducting health examinations. In order to evaluate the effects of the AAT, the authors elected to carry out standard tests applied in physiotherapy and nursing care. In terms of physiology, values for blood pressure (diastolic and systolic) and heart rate were obtained. These measurements were conducted every third day, always in the morning, after breakfast and prior to therapy.

Self-sufficiency was evaluated by applying the Barthel index, wherein any independent activities undertaken were considered [22,23]; these were conducted and assessed in accordance with the standard procedures of the hospital. The patients underwent this test at the commencement of the six-week cycle of therapy and upon its cessation (pre and post tests). This index ranged from 0–100.

For evaluating the mood, the replies given by the members of the cohort were assessed in accordance with a Likert scale, which enabled a clarification of the content and recorded the approximate strength of reaction [24,25]. It comprised a ten-point scale, herein applied to describe the mood of the individual on the given day. The members of the experimental and control group were asked the question “How do you feel today?”, which was gauged on a scale of 1 to 10, with “1” being the worst possible reaction (not at all well) and “10” the best (extremely well). Patients in the experimental group were interviewed at the start and end of each session, while the control group patients were also asked said question on the same days twice a week. From every patient we had 12 evaluations of mood taken before and after sessions.

### 2.5. Data Analysis

The authors carried out exploratory data analysis to verify assumptions that would act as a baseline for subsequent processing (such as sampling independence, homogeneity, and normal distribution). Their hypothesis utilized the mechanism of a Gaussian distribution, which was assessed using the Shapiro–Wilk test, and further certified using histograms and normalized probability diagrams.

The frequencies of data for the given patients differed. The initial value (the status prior to commencement of therapy) was stipulated as the median, which was derived from the first five measurements, while the median from the last five measurements was designated as the final value (after therapy had finished).

Data analysis indicated a Gaussian distribution in most cases, although, in light of the limited quantity of respondents, a very slight deformation from normality was obtained. Parametric tests were carried out to evaluate generalization and the significance of differences in the values obtained. In order to evaluate the variance between values for the commencement and close of the study period, a paired t-test was applied for dependent samples. Variations in the results recorded for the experimental and control groups were analyzed using a two-sample t-test and F-test. Moreover, contingency tables were drawn up, within which the patients were classified according to their condition after the therapy had ceased, as follows: (i) showing improvement, (ii) showing deterioration, or (iii) unchanged. It was necessary for there to be a minimum of a 10% difference in results between the commencement and close of therapy to changed type of classification; the Barthel scale was employed for calculation purposes. Gauging the significance of values for differences and generalization was possible by applying Pearson’s chi-squared test. The degree of the relationship was determined using the value for the contingency coefficient (C). This test was also conducted in order to derive the extent of variation in the subjective mood of the patients as to their outlook baseline. Statistical significance was set at the level of *p* < 0.05.

Additionally, the contingency tables contained odds ratios (ORs), which were evaluated and interpreted in order to summarize connections between the associated frequencies. The odds ratios constituted a multiplicative approach to assessment. Notably, the opportunity arose in the present study to address the following question: “How likely is improvement in the physical and psychological condition of the patient if a dog is present in therapy sessions?”.

## 3. Results

**Figure 1:**
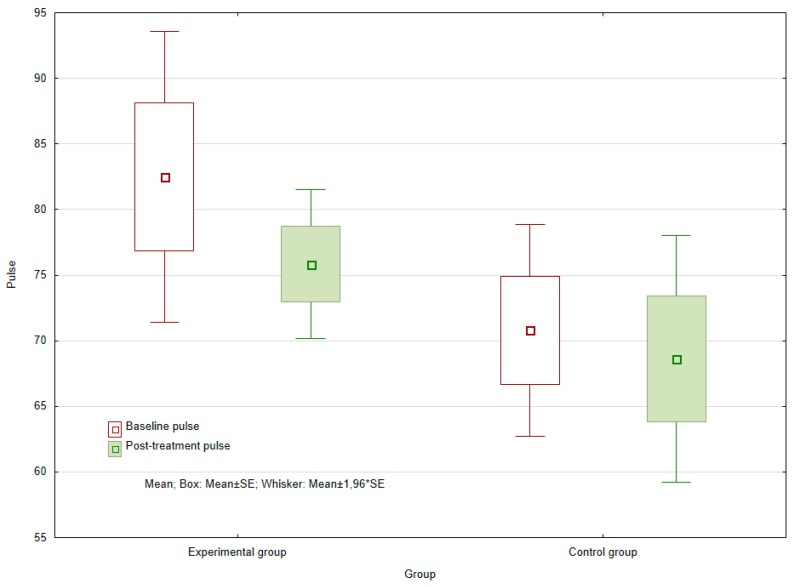
Baseline and post-treatment heart rate of the experimental and control groups.

The results show that no statistically significant difference was demonstrated in the control group (*p* = 0.957) between median heart rate values before and after six week of observed period. In the experimental group, no statistically significant difference was evident between the baseline and the post-treatment values for heart rate. In any case, the significance level of *p* = 0.08894 still remained very low. Due to the limited number of patients involved in the experimental group (*n* = 6) and the minimal variation in the values obtained, no reduction in heart rate after completion of the six-week course of therapy could be statistically proven. However, given more observations, it would be possible to assume that such differences could still be rejected (see Figure 1).

**Figure 2:**
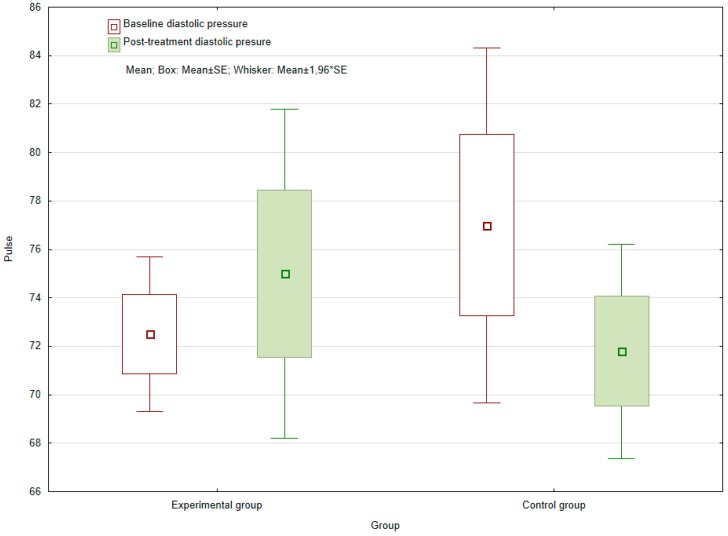
Baseline and post-treatment diastolic blood pressure of the experimental and control groups.

Within diastolic blood pressure measurements prior to and following the cycle of therapy, a surprising statistically significant decrease in the control group was observed (*p* = 0.0128), which was not witnessed in the experimental group (*p* = 0.519). No statistically significant difference in mean values between the control and experimental groups was demonstrated at baseline, although comparing the final values revealed a statistically significant difference between the groups (*p* = 0.0102) (see Figure 2). In the experimental group, blood pressure values were closer to 80 mmHg, whereas in the control group, the values were lower, at approximately 70 mmHg. However, both groups exhibited variance in values for physiological status.

For systolic blood pressure, no statistically significant differences in baseline and endpoints were found in either group. Additionally, no statistically significant difference was observed in baseline and final values between the experimental and control groups.

**Figure 3:**
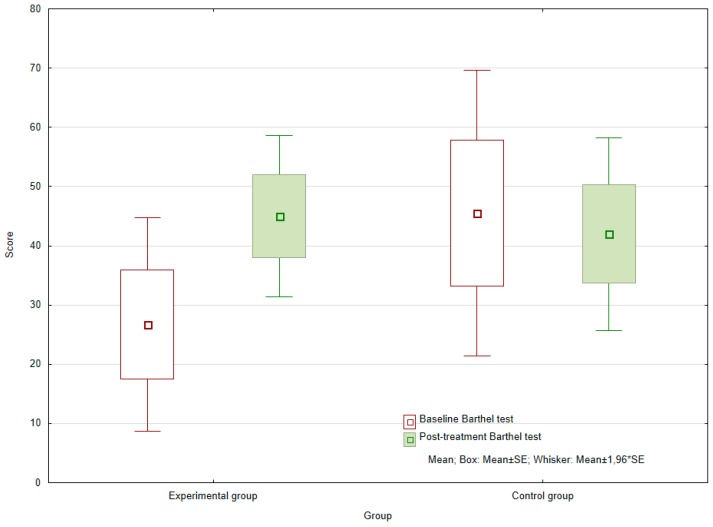
Baseline and post-treatment Barthel index scores of the experimental and control groups.

When comparing the initial and final values for the Barthel index, i.e., the rating for self-sufficiency, no statistically significant difference was found in the control group (*p* = 0.7865). In contrast, values for the experimental group showed a significant increase, pointing to improved patient self-sufficiency (*p* = 0.0060) (see Figure 3). Within the experimental group, the average absolute difference between the input and the final value was 18.333. 

The difference in Barthel index values for the experimental and control groups at the baseline (*p* = 0.2006) and the end (*p* = 0.6197) of the observed period were not statistically significant. However, the chances of achieving a better score for this index were 3.3 times greater for those who had received AAT therapy than the control group (OR = 3.333). 

Subjective assessment of the well-being of the patients was based upon the feedback they gave on their mood before and after each therapy session (every patient 12 sessions), measured via a 10-point Likert scale. This parameter of mood was also gauged prior to commencing the study and upon its close. The values reported during the research period (always the average of three consecutive measurements) showed a positive shift in both the experimental (*p* = 0.02771) and the control (*p* = 0.0077) groups. Nobody reported a decrease of mood.

**Figure 4:**
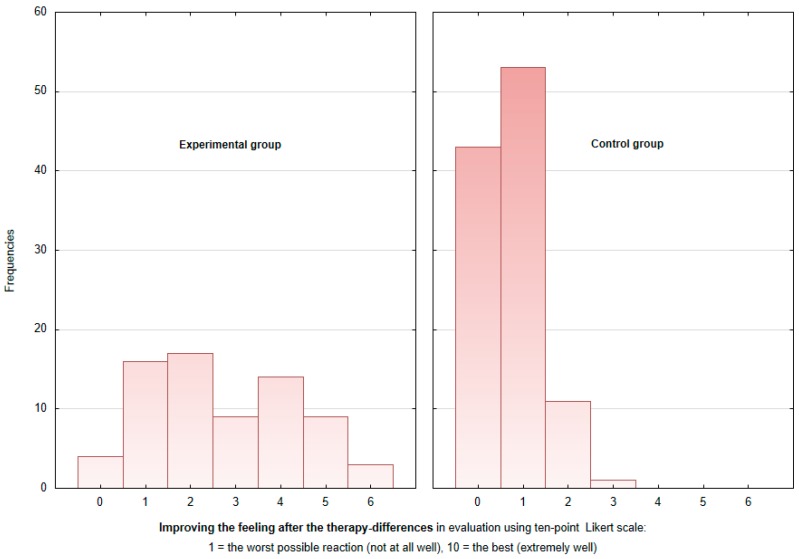
Subjective assessment of the well-being differences between pre- and post-session in the experimental and control groups.

Interestingly, the subjective assessment of mood given by the patients was strongly dependent upon the type of therapy they had received, with the experimental group responding more positively (C = 0.5594, at the confidence level of 95%). Figure 4 shows that while in the control group, mood changes were predominantly +1 and unchanged on the Likert scale, in the experimental group, mood changes were almost evenly divided between +1 to +5, with mood changes of +2, +1, and +4 points on the Likert scale being most common in decreasing order. While in the control group, none of the patients declared a mood change after therapy of 4 to 6 points, the experimental group reported such mood improvement after therapy. The mean sense of improvement after AAT equaled +3.5 points (above the median) for the experimental group and +1 point in the control group (see Figure 4).

## 4. Discussion

Since this is a pilot study and the number of patients involved is limited, the authors do not find it appropriate to generalize the results. The cohort should be larger to confirm the findings; therefore, it would be useful for future studies to include more patients, given that the results in the current study are promising for a positive outcome/effect of AAT. Even so, we find it interesting that some of our results do not coincide with previous studies. This happened with the AAT effect on the heart rate and the blood pressure. Given the number of studies confirming the reduction of these parameters after AAT, it could be assumed that this effect is already well described [10,26] and is connected with a decrease of the cortisol level and a consequent drop in stress [27,28,29,30]. Reduction in blood pressure has also been observed in studies by Allen et al. [31,32] and Allen [33]. Therefore, it seems that it depends very much on the conditions in which this effect occurs, such as the time of measurement, the length of stay of the animal, the ownership of the animal, etc.

Herein, no statistically significant difference between the experimental and the control groups was seen in values for heart rate, although the significance level for the former of the two was low, suggesting the dog had exerted some kind of effect on the patients and a larger sample of observations could affect this result.

No discernible effect of AAT was observed in the values for blood pressure. This was inconsistent with the studies mentioned above, although the cohorts therein comprised patients with elevated blood pressure, where a drop in blood pressure meant a shift toward the norm. In contrast, the values for both groups in the present study ranged between 60–90 mmHg. Blood pressure is influenced by a number of factors, including the activity of the patient in question [34]. Therefore, the higher values for diastolic pressure recorded for the experimental group comparing the values of control group might have been associated with the greater physical activity performed by the patients through the contact with the dog. An alternative reason could be that the methodology adopted herein for evaluating this parameter differed from those of other studies.

Divergence between the two groups was observed in the values for the Barthel index. However, the shift seen in the experimental group may have been initiated through more than just partaking in exercise and activity with the dog. Due to the frequency of visits, such interactions proved to be rather pleasant experiences for the individuals, and a possible means of overcoming any fear of failure. The improvement observed might also be explained by the better psychological state of the experimental group, reflected through its members demonstrating heightened co-operation with other therapists and additional motivation.

Comparing the input and output ratings of the subjective feeling of well-being for the experimental and control patients revealed a statistically significant improvement in both, indicating that everybody had received an effective course of rehabilitation for their health problems. The major difference was primarily seen in the change in mood during individual therapy sessions, as the experimental group expressed a more pronounced variation between feedback given before and after each session than the control group, who had participated in the standard departmental program. Indeed, an improvement in the mood of patients who had interacted with dogs was also reported in another study [35].

The advantages of this study are that it took place in an actual hospital, wherein the patients stayed under the rehabilitation process, and it was integrated as a supporting part of the comprehensive approach. After clarifying all and any conditions, safety measures, and other matters that were necessary, the course of the study was properly incorporated into the rehabilitation schedule for the patients, and carried to completion. Its findings go beyond highlighting the improved state of mind of the individuals to demonstrate how AAT can be implemented at a hospital. 

## 5. Limitations

The primary constraint is the limited number of patients in this study. Since this constitutes a pilot study, these initial results represent the starting point for additional research, as it has highlighted the feasibility of incorporating AAT into the therapeutic treatment of stroke patients. It should be possible to build on the research conducted and therefore further develop the notion of a dog (or another domesticated animal) being present at rehabilitation sessions for patients as appropriate. Another potential limitation is the medication the individual received, which could have affected the blood pressure measured; this is important with respect to ill patients such as these, since their blood pressure is managed to keep it as stable as possible. An additional limitation is the individual therapeutic approach required for each patient involved. Lastly, the personality of the therapist and the dog may have affected the outcome of the therapy. 

## 6. Conclusions

The results of the study confirmed that, at a subjective level, patients who received AAT felt better, although changes in their values for heart rate and blood pressure remained inconclusive. Several factors that could benefit therapy were highlighted through this research, which were the positive rehabilitation of patients, the pro-active approach they adopted to treatment, their subsequent reciprocity with other therapists, and the progression in relationships with attending personnel. Incorporating AAT, with the aid of a therapy dog, into the rehabilitation process of patients would appear to be a feasible and recommended step to take.
